# Dissecting the RecA-(In)dependent Response to Mitomycin C in *Mycobacterium tuberculosis* Using Transcriptional Profiling and Proteomics Analyses

**DOI:** 10.3390/cells10051168

**Published:** 2021-05-11

**Authors:** Anna Brzostek, Przemysław Płociński, Alina Minias, Aneta Ciszewska, Filip Gąsior, Jakub Pawełczyk, Bożena Dziadek, Marcin Słomka, Jarosław Dziadek

**Affiliations:** 1 Institute of Medical Biology of the Polish Academy of Sciences, Lodowa 106, 93-232 Łódź, Poland; przemyslaw.plocinski@gmail.com (P.P.); alinagorna@gmail.com (A.M.); kanetka1@tlen.pl (A.C.); filip.gasior95@gmail.com (F.G.); jpawelczyk@cbm.pan.pl (J.P.); 2 Department of Immunology and Infectious Biology, Faculty of Biology and Environmental Protection, University of Łódź, Banacha 12/16, 90-237 Łódź, Poland; 3 Department of Molecular Microbiology, Faculty of Biology and Environmental Protection, University of Łódź, Banacha 12/16, 90-237 Łódź, Poland; bozena.dziadek@biol.uni.lodz.pl; 4 Biobank Lab, Department of Molecular Biophysics, Faculty of Biology and Environmental Protection, University of Łódź, Pomorska 139, 90-235 Łódź, Poland; marcin.slomka@biol.uni.lodz.pl

**Keywords:** DNA damage repair, SOS response, tuberculosis

## Abstract

Mycobacteria exploit at least two independent global systems in response to DNA damage: the LexA/RecA-dependent SOS response and the PafBC-regulated pathway. Intracellular pathogens, such as *Mycobacterium tuberculosis*, are exposed to oxidative and nitrosative stress during the course of infection while residing inside host macrophages. The current understanding of RecA-independent responses to DNA damage is based on the saprophytic model of *Mycobacterium smegmatis*, a free-living and nonpathogenic mycobacterium. The aim of the present study was to identify elements of RecA-independent responses to DNA damage in pathogenic intracellular mycobacteria. With the help of global transcriptional profiling, we were able to dissect RecA-dependent and RecA-independent pathways. We profiled the DNA damage responses of an *M. tuberculosis* strain lacking the *recA* gene, a strain with an undetectable level of the PafBC regulatory system, and a strain with both systems tuned down simultaneously. RNA-Seq profiling was correlated with the evaluation of cell survival in response to DNA damage to estimate the relevance of each system to the overall sensitivity to genotoxic agents. We also carried out whole-cell proteomics analysis of the *M. tuberculosis* strains in response to mitomycin C. This approach highlighted that LexA, a well-defined key element of the SOS system, is proteolytically inactivated during RecA-dependent DNA repair, which we found to be transcriptionally repressed in response to DNA-damaging agents in the absence of RecA. Proteomics profiling revealed that AlkB was significantly overproduced in the Δ*recA pafBC*^CRISPRi/dCas9^ strain and that Holliday junction resolvase RuvX was a DNA damage response factor that was significantly upregulated regardless of the presence of functional RecA and PafBC systems, thus falling into a third category of DNA damage factors: RecA- and PafBC-independent. While invisible to the mass spectrometer, the genes encoding *alkA*, *dnaB*, and *dnaE2* were significantly overexpressed in the Δ*recA pafBC*^CRISPRi/dCas9^ strain at the transcript level.

## 1. Introduction

Tuberculosis (TB) is caused by the intracellular pathogen *Mycobacterium tuberculosis* (*M. tuberculosis*), and it remains a serious bacterial threat to global health because strains resistant to drugs currently used in the clinic are increasingly emerging [1]. Genomic integrity is critical to the survival and proliferation of the bacterium. While residing within host macrophages, the bacterium is exposed to the DNA-damaging action of oxygen and nitrogen radicals produced by macrophages as a natural response to infection [2]. DNA damage can also be triggered directly or indirectly by chemical substances used to treat the infection as well as by the hypoxic conditions within the granuloma [3]. On the other hand, unfaithful, error-prone DNA repair is one of the main factors leading to mutation, which can lead to the selection of strains carrying mutations responsible for drug or multidrug resistance. A balance between DNA repair and mutation is achieved by multiple levels of regulation of various DNA repair pathways. Negligible DNA damage is repaired by adequate error-free mechanisms, while exposure to a serious source or level of DNA damage induces more error-prone mechanisms [4]. Such a response is necessary when potentially deadly blocking lesions are created or alternative repair pathways are inefficient or ineffective with specific types of damage. This includes double-strand DNA breaks in nonreplicating bacteria as well as any damage that requires translesion synthesis when the DNA damage has to be tolerated to proceed with a round of DNA replication necessary for proliferation [5].

*M. tuberculosis* possesses a suite of mechanisms responsible for orchestrated repair of various types of DNA damage. Homologous recombination, nonhomologous end joining, base and nucleotide excision, and translesion DNA synthesis are all operational in mycobacterial cells. Until quite recently, it was believed that the bacterium does not encode any mismatch repair system; however, this hypothesis was nullified with the discovery of NucS endonuclease, an archaeal-like mismatch repair enzyme [6]. Among other unique features of the mycobacterial DNA repair machinery is the enormously branched and exaggerated base excision repair (BER) system [7]. These and other features are likely responsible for the very high genomic stability of mycobacterial genomes.

One of the most important factors involved in the repair of DNA damage in mycobacterial cells is the RecA protein. Recombinational repair conferred by the activity of RecA is central to the exchange of a damaged DNA strand, while a second undamaged copy is present inside the same cell. RecA recognizes DNA strand homology and initiates strand migration in the process of homologous recombination (HR) [2]. Mycobacterial RecA forms filaments on DNA and cooperates closely with the single-stranded DNA-binding protein SSB, which stimulates RecA activity and promotes strand exchange. This mechanism is important for the RecBCD- and RecFOR-mediated pathways, both of which require RecA for strand invasion. In mycobacteria, single-strand breaks are preferentially processed by RecFOR or RecOR [8] systems, whereas the AdnAB pathway specializes in sealing double-strand breaks [9]. The *M. tuberculosis* RecA protein is also known to interact with the UvrD1 and UvrA proteins, which are involved in nucleotide excision repair (NER) [10]. The activities of RecA are not limited to DNA strand exchange, as it also acts as an important coregulator of the SOS response. RecA filaments forming on DNA induce the autocatalytic activity of the LexA regulator, which in turn no longer represses the expression of a set of proteins related to DNA repair [11]. The expression of the *recA* gene is driven by two promoters that can induce DNA damage. One of the two promoters contains a LexA binding consensus and was shown to be regulated by the LexA repressor [12]. The RecA protein is thus an effector as well as a sensor of DNA damage in the LexA/RecA-mediated SOS response.

The two main models used to study SOS responses in bacteria include DNA damage caused by exposure to damaging doses of UV light or exposure to mitomycin C. Previous studies on *Mycobacterium* (*Mycolicibacterium*) *smegmatis* (*M. smegmatis*), a saprophytic cousin of *M. tuberculosis*, discovered PafBC, an unusual transcription factor complex that regulates a large number of genes in response to mitomycin C via a LexA-independent route [13]. The RecA/LexA-mediated and PafBC-regulated regulatory pathways control a gross number of genes during mitomycin C-induced DNA damage [14]. The present study was designed to excerpt proteins involved in the orchestra of factors responding to mitomycin C damage in pathogenic *M. tuberculosis*.

## 2. Materials and Methods

### 2.1. Bacterial Strains Cultures

*E. coli* strains were cultured for 18–20 h at 37 °C in liquid or solid Luria–Bertani medium supplemented if required with 50 μg/mL kanamycin (Bioshop, Burlington, ON, Canada) or 100 μg/mL ampicillin (Bioshop, Burlington, ON, Canada). The *M. tuberculosis* cultures were carried out in 7H9 or 7H10 broth (Difco, Baltimore, MD, USA) with OADC (oleic acid, albumin, dextrose, catalase; Difco, Baltimore, MD, USA) and 0.05% Tween 80 (Sigma Aldrich, St. Louis, MO, USA) at 37 °C and were supplemented with antibiotics or other ingredients, if necessary, at the following concentrations: kanamycin (Sigma Aldrich, St. Louis, MO, USA), 25 μg/mL; anhydrotetracycline (aTc; Sigma Aldrich, St. Louis, MO, USA), 100 ng/mL; or mitomycin C (Sigma Aldrich, St. Louis, MO, USA), 5 ng/mL.

A list of *M. tuberculosis* strains used in this study is presented in Supplementary Table S1. To determine the growth rates, bacterial cells were cultured to an OD_600_ of 1.0 in 7H9/OADC/Tween 80 medium. Then, seed cultures were used to inoculate fresh 7H9 broth supplemented with OADC/Tween 80 at an OD_600_ of 0.05. The cultures were incubated at 37 °C for 5–14 days. At 24 h or 48 h intervals, the samples of cultures were analyzed using a spectrophotometer (Pharmacia Biotech Ultrospec 2000, GE Healthcare, Uppsala, Sweden). To assess the number of CFUs (colony forming units), samples were serially diluted in fresh 7H9/OADC/Tween 80 broth, plated on 7H10/OADC/Tween 80 medium and incubated at 37 °C for 3–4 weeks. Each experiment was performed at least in triplicate.

*M. tuberculosis* mutants. The *Mtb* knockout mutant was obtained by using a gene replacement protocol as previously described [15]. To generate a knockdown (KD) strain depleted of PafB (Rv2096c) and PafC (Rv2095c) proteins, which are a part of the Pup-proteasome system (PPS), the CRISPRi/dCas9 strategy based on the pLJR965 plasmid was applied [16]. The gene-specific sgRNA probes carrying approximately 20 nucleotide-long target sequences appropriately spaced from the PAM site (Supplementary Table S2) were planned according to the published protocol and cloned into the pLJR965 plasmid, which was introduced into the *M. tuberculosis* H_37_Rv laboratory strain. The resulting recombinant strains carrying dCas9 and sgRNA were verified by PCR using the appropriate primers Crispr/Cas9-F and Crispr/Cas9-R (Table S2). The efficacy of silencing was monitored by the growth kinetics of tested strains in the presence of anhydrotetracycline at a concentration of 100 ng/mL and compared to the culture of *M. tuberculosis* carrying “an empty” pLJR965 plasmid. For total RNA sequencing, the strains were incubated under the above-mentioned conditions for 7 days, cells were then harvested by centrifugation, and total RNA was isolated.

### 2.2. Recombinant M. tuberculosis RecA Expression, Purification, and Production of Anti-RecA Rabbit Polyvalent Serum

The *recA_Mtb_* gene (*rv2737c*) was amplified by PCR (primers listed in Supplementary Table S2) and introduced into the pHIS parallel expression plasmid [17] with 6-HIS *N*-terminal fusion. The resulting plasmid was introduced into the *E. coli* BL21 expression strain. Protein expression was performed at 15 °C for 4 h in the presence of 0.4 mM IPTG. The pellet was harvested by centrifugation and resuspended in 10 mL of binding buffer (50 mM Tris-HCl, pH 8.0, and 6 M urea, Sigma Aldrich, St. Louis, MO, USA) and sonicated in short bursts (Bioblock Scientific Vibracell). Next, the sample was incubated for 2 h at room temperature and then centrifuged at 17,000× *g* at 12 °C for 30 min, and the supernatant was placed on an affinity column containing Ni-NTA resin (Thermo Fisher Scientific, Waltham, MA, USA) to bind the protein. Next, the column was washed with binding buffer and wash buffer (60 mM imidazole, 0.4 M NaCl, 20 mM Tris-HCl, pH 8, and 6 M urea, Sigma Aldrich, St. Louis, MO, USA). Next, the recombinant protein was washed out with elution buffer (1 M imidazole, 0.5 M NaCl, 20 mM Tris-HCl, pH 8, and 6 M urea, Sigma Aldrich, St. Louis, MO, USA). The recombinant protein was concentrated on a Novagen concentrator to a final concentration of 1 mg/mL and then used to immunize New Zealand rabbits raised under standard conventional conditions, which were approved by the Polish Ministry of Science and Higher Education Animal Facility of the Institute Microbiology, Biotechnology and Immunology, Faculty of Biology and Environmental Protection, University of Lodz. The experimental procedures were approved and conducted according to guidelines of the appropriate Polish Local Ethics Commission for Experiments on Animals No. 9 in Lodz (Agreement 9/ŁB87/2018). The immunization protocol consisted of three subcutaneously injected doses (dose I, 250 µg; doses II and III, 200 µg in 0.5 mL of PBS) of recombinant RecA emulsified with an equal volume of IFA (Incomplete Freund’s Adjuvant) in 3-week intervals followed by the procedure published for PPE51 protein [18].

### 2.3. Total Protein Isolation and Western Blotting

*M. tuberculosis* cell lysates were prepared by bead beating (using 0.1 mm zirconia beads) and used for immunodetection using polyclonal antibodies raised against RecA (this study) and LigA proteins [19]. The total protein concentration was determined by the Bradford method (Sigma Aldrich, St. Louis, MO, USA). To compare the amount of RecA protein in various samples, equal concentrations of the total protein were separated in sodium dodecyl sulfate-polyacrylamide gels, transferred to nitrocellulose membranes, immunodetected with anti-RecA and anti-LigA polyclonal antibodies using the Amersham Pharmacia ECL chemiluminescence kit and protocol, and visualized on Hyperfilm ECL (GE Healthcare, Uppsala, Sweden Amersham Pharmacia Biotech UK Ltd.).

### 2.4. Survival of M. tuberculosis Strains Exposed to UV Light or in the Presence of Mitomycin C (MMC)

The survival of *M. tuberculosis* strains was assessed in 7H9/OADC/Tween 80 medium supplemented with 5 ng/mL MMC (wild-type and Δ*recA*) and 100 ng/mL aTc (control CRISPR-Cas9 strain and mutants: *pafBC*^CRISPRi/dCas9^ and Δ*recA pafBC*^CRISPRi/dCas9^) at an OD_600_ of 0.05 using standard CFU methodology at the following time points: 0 h, 48 h, 96 h, and 168 h. Serial 10-fold dilutions of cells were plated on 7H10/OADC/glycerol and incubated for 3–4 weeks at 37 °C. Next, colonies were counted and a *t*-test was applied to determine the statistical significance between the test and control values. The growth kinetics were monitored by measuring the optical density OD_600_ at 0 h, 48 h, 96 h, and 168 h after the addition of MMC.

Wild-type *M. tuberculosis* and its mutants were grown to the logarithmic (OD_600_ of 0.8) or stationary phase (14 days, reaching an optical density OD_600_ of 2.0) in 7H9 liquid media supplemented with OADC and 100 ng/mL anhydrotetracycline (control CRISPR-Cas9 strain and mutants: *pafBC*^CRISPRi/dCas9^ and Δ*recA pafBC*^CRISPRi/dCas9^). Next, the cells were serially diluted, and 100 μL of each cell suspension (10-fold diluted) was spread onto 7H10 solid agar supplemented with OADC and glycerol. Then, the plates were treated with UV at doses of 5 mJ, 10 mJ, and 15 mJ and incubated at 37 °C for 3–4 weeks. The control set of plates was not treated with UV. After incubation time, the colonies were counted and a *t*-test was used to determine the statistical significance between the test and control values. Each experiment was performed at least in triplicate.

### 2.5. RNA Isolation and Sequencing

For RNA isolation, the *M. tuberculosis* wild-type strain and its Δ*recA* mutant were grown in a rich medium supplemented or not with 5 ng MMC at an OD_600_ of 0.05. The control *M. tuberculosis* strain carrying the CRISPR-Cas9 integrative plasmid and the mutant *pafBC*^CRISPRi/dCas9^ were grown in a rich medium supplemented with anhydrotetracycline (100 ng/mL) until OD_600_ = 0.8. The cultures were then refreshed with a fresh medium, and the new cultures were supplemented with both anhydrotetracycline (100 ng/mL) and MMC (5 ng/mL) at an OD_600_ of 0.1 and incubated in roller bottles at 37 °C. At OD_600_ = 0.4–0.8, cells were spun down, and the bacterial pellet was lysed by bead beating with the MP FastPrep system using TRIzol LS reagent (Thermo Fisher Scientific, Waltham, MA, USA) as described previously [20].

The DNA contamination of RNA samples was removed by treatment with DNase I turbo (Thermo Fisher Scientific, Waltham, MA, USA) following the manufacturer’s protocol. The quality of RNA samples was examined using an Agilent 2100 BioAnalyzer and the standard protocol (Agilent RNA 6000 Nano Kit, Agilent Technologies, Inc., Santa Clara, CA, USA). The Illumina-compatible RNA/cDNA libraries were prepared according to the detailed description provided in our previous study [21]. Before the preparation of the sequencing libraries, ribosomal RNA was removed with a Ribo-Zero rRNA Removal Kit (Illumina Inc., San Diego, CA, USA) and purified with AMPure XP magnetic beads (Becton Dickinson, New York, NY, USA). The KAPA Stranded RNA-Seq kit (KAPA Biosystems LTD, MA, USA, Cape Town, South Africa) and Illumina True Seq v2 indexing system were applied to prepare Illumina-compatible RNA/cDNA libraries, which were evaluated on an Agilent 2100 BioAnalyzer fitted with a DNA 1000 chip. On average, 6–12 million paired-end reads per sample were obtained with the NextSeq500 System (Illumina Inc., San Diego, CA, USA) and the NextSeq 500/550 Mid Output v2 sequencing kit (150 cycles, Illumina Inc., San Diego, CA, USA).

### 2.6. Transcriptional and Proteomics Data Analysis

The processing of RNA sequencing data was completed with a series of software and scripts as described in [21]. Briefly, adapter-free reads [22] with a minimal length of 20 bp and a minimum quality of 30% (Sickle script) were aligned to the genome of *M. tuberculosis* H_37_Rv (NC_018143.2) with the Bowtie2 short read aligner [23]. The SAMtools software suite was used for data handling, converting, and indexing [24]. The level of global expression in the analyzed samples was compared using the default parameters of the online Degust RNA-Seq analysis platform [25]. The false discovery rate (FDR) represented the statistical analysis of differential gene expression (DGE) calculated by Degust. Differential gene expression was called when FDR < 0.05 and the log2-fold change > |1.585| (changing three times or more).

The mass spectrometry analyses of the whole-cell protein lysates obtained according to the previously published methodology [21] were performed as a service at the Institute of Biochemistry and Biophysics PAS on a Q Exactive high-performance mass spectrometer using an experimental pipeline reported elsewhere [26].

### 2.7. Evolutionary Pressure Analysis

We used cooccurrence gene detection of STRING Protein software to identify gene homologs across the phylogenetic tree of life. We estimated the variability of gene sequences across a population of 3978 clinical strains of *M. tuberculosis* [27]. The reference sequence to estimate SNP variations was *M. tuberculosis* H37Rv (NC_000962). SNP variations were assessed with Geneious Prime software (Biomatters LTD, Auckland, New Zealand) [28]. DNA variability was estimated with DnaSPv6 [28]. We quantified the ratios of nonsynonymous and synonymous nucleotide substitution rates (dN/dS) on a per-site basis for a given coding alignment and corresponding phylogeny with MEGA7 [28].

## 3. Results

### 3.1. DNA Repair Genes under Evolutionary Pressure

Homologs of selected DNA repair genes (*alkA, dnaB, rv2554c/ruvX, uvrA, pafB, pafC, recA, lexA, dnaE2,* and *ruvA*) of the RecA-dependent and RecA-independent pathways were identified in various organisms across three kingdoms of the phylogenetic tree of life (Supplementary Figure S1). The most widespread homolog present abundantly in Bacteria, Eukaryota, and Archaea was *recA*. The presence of *lexA* was restricted to bacteria and episodically to Viridiplantae and Opisthokonta. The second-most widespread homolog among all genes analyzed was *uvrA*. *uvrA* underwent significant sequence divergence in the Eukaryota kingdom but showed relatively high sequence homology in archaea and bacteria. The least distributed genes were *pafB* and *pafC*, which were restricted to certain species of the Bacteria kingdom, where they show relatively low sequence conservation.

Furthermore, the variability of the selected genes was assessed across the 3978 *M. tuberculosis* clinical strains (Supplementary Table S3). We retrieved the complete gene sequences from 99.39% (*n* = 3775) to 100% (*n* = 3798) of strains depending on the gene. We observed rare deleterious frameshift mutations in six genes: *dnaB, dnaE2, lexA, pafC, recA, ruvA*, and *uvrA*. Strains carrying mutations leading to deleterious mutations represented a minuscule fraction of the population ranging from 0.03% (*n* = 1) for *pafC* to 0.16% (*n* = 6) for *dnaE2*. However, some of the observed frameshifts were located in single-nucleotide tandem repeat regions, suggesting possible sequencing errors. After we stripped the alignment of sequences carrying insertions/deletions, we estimated that the genes contained on average 3023 polymorphic sites per 100 bp. The most variable gene was *lexA*, which contained 4078 polymorphic sites per 100 bp. The most conserved gene across the population was *recA*, with 1769 polymorphic sites per 100 bp. The *M. tuberculosis* evolutionary pressure level on each gene varied considerably (range 0.321–1.745) (Supplementary Table S4). Overall, the dN/dS ratio across all selected genes was 0.867 ± 0.384. *ruvA* and *ruvX* showed the lowest and highest dN/dS ratios, respectively.

### 3.2. Deficiency of RecA and/or PafBC Leads to the Sensitization of Tubercle Bacilli to DNA Damage

**Figure 1:**
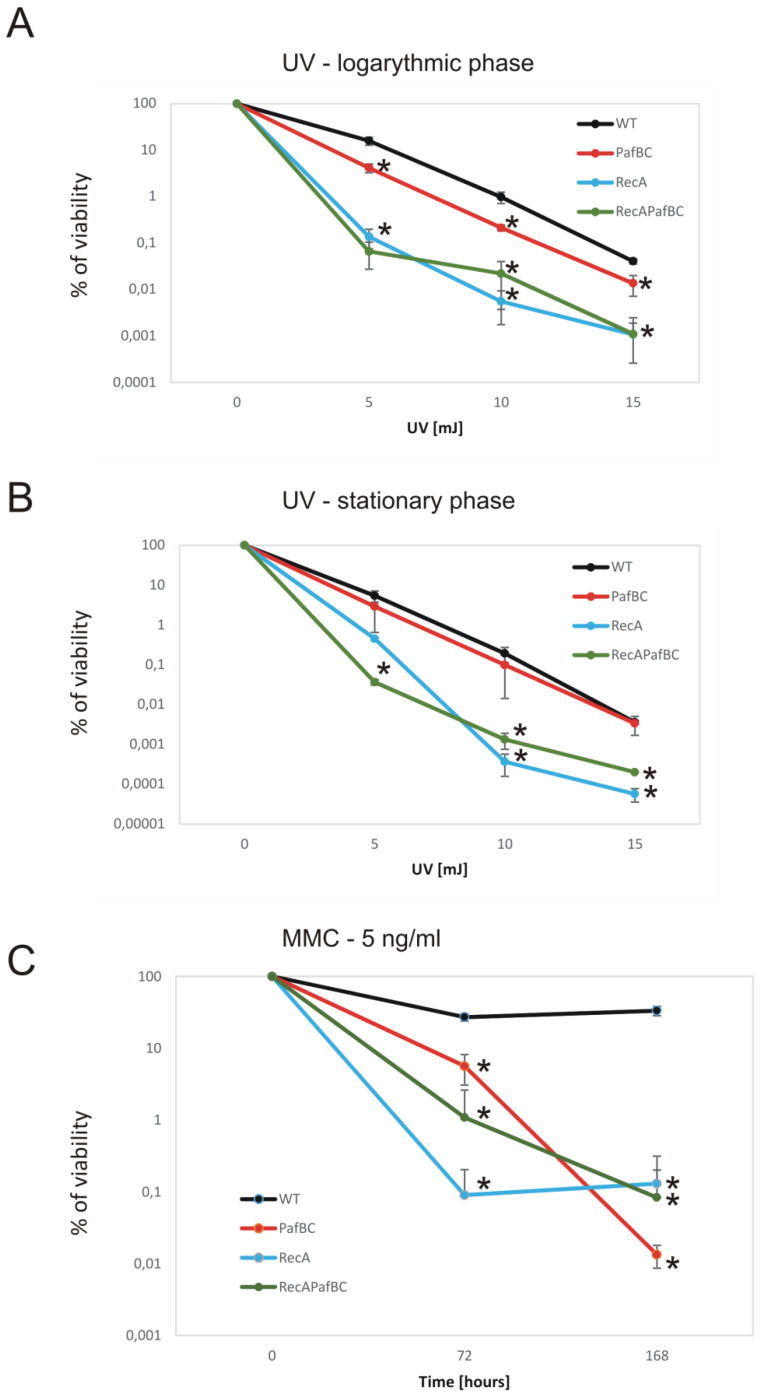
Viability of *M. tuberculosis* mutants exposed to UV and MMC. The viability of wild-type *M. tuberculosis* (WT) and its mutants (RecA-Δ*recA*; PafBC-*pafBC^CRISPRi/dCas9^*; RecA PafBC-Δ*recA*-*pafBC^CRISPRi/dCas9^*) exposed to 5, 10, or 15 mJ UV (**A**,**B**) or 5 ng/mL MMC for 72 and 168 h (**C**) based on CFU analysis. The percentage of viability was calculated by comparing the number of viable cells in treated vs. untreated samples from at least three independent experiments and plotted as the average ± standard deviation. A *t*-test was employed for comparisons of mutants versus the control samples (WT) to determine any significant differences between the mean values of the wild-type and mutant strains. The results were considered statistically significant (*) at *p* < 0.05.

As identified in the *M. smegmatis* model strain, the response to DNA damage in mycobacteria is regulated by (i) the LexA/RecA-dependent SOS response and (ii) the PafBC RecA-independent pathway [13,14]. It was also reported that PafBC-deficient mutants are sensitized to mitomycin C and UV radiation and that RecA overproduction induced by DNA damage is partially regulated by PafBC [13]. Here, we verified whether the correlation between the LexA/RecA system and PafBC is also present in pathogenic *M. tuberculosis* strains. The identity between RecA of *M. tuberculosis* and *M. smegmatis* is as high as 90%; however, the RecA of *M. tuberculosis* contains an intein that is absent in RecA of *M. smegmatis*. PafB and PafC of *M. tuberculosis* and *M. smegmatis* share 46.8% and 69.5% identity, respectively (Figure S2). We compared the DNA damage sensitivity of the RecA-deficient *M. tuberculosis H37Rv* strain (Δ*recA*) constructed by gene replacement [15] to the mutant depleted of PafBC using the CRISPR-Cas9 system, and to the double mutant strain deficient in the synthesis of RecA and depleted of PafBC, which were described in the Materials and Methods section. As expected, the Δ*recA M. tuberculosis* mutant appeared to be very sensitive to UV radiation (Figure 1A,B). The viability of Δ*recA* decreased significantly (*p* < 0.05) compared to the wild-type *Mtb* and to the CRISPRi/dCas9 mutant depleted of PafBC after exposure to 5, 10, and 15 mJ UV radiation, in both stationary and logarithmic phases of growth. The depletion of PafBC sensitized the mutant strain to UV light compared to the wild-type strain (*p* < 0.05) in the logarithmic but not the stationary phase of growth. The depletion of PafBC in the Δ*recA* mutant did not significantly affect the UV sensitivity of the RecA-deficient strain. The synergistic effect was also not observed under treatment with 5 ng/mL mitomycin C. The viability of a single Δ*recA* mutant decreased at the 72 h time point significantly more than *pafBC^CRISPRi/dCas9^* (*p* < 0.05), but at the 168 h time point all mutants were sensitized to MMC at similar levels (Figure 1C).

**Figure 2:**
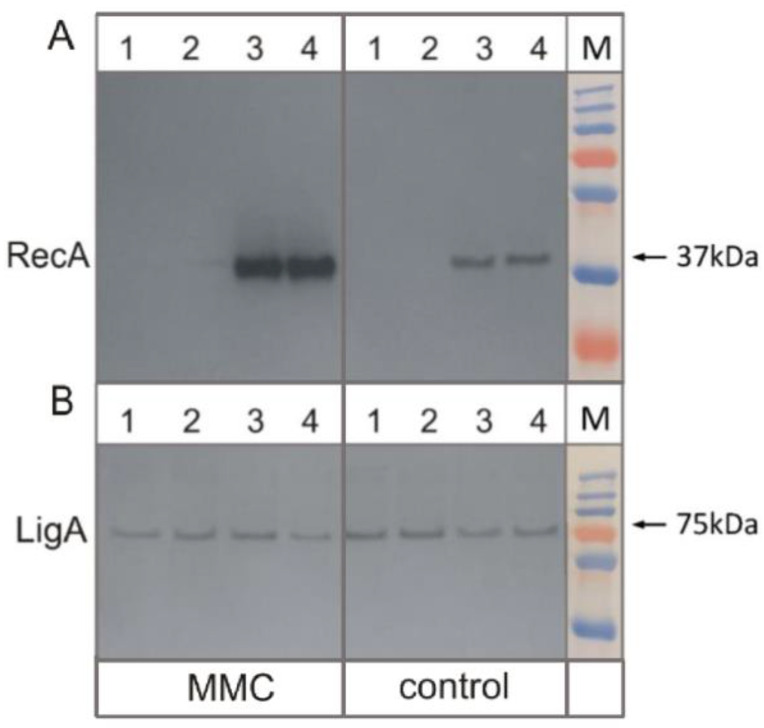
Immunodetection of RecA protein in *M. tuberculosis* mutant cell lysates. The levels of RecA (panel A) and control protein LigA (panel B) in the wild-type strain (lanes 4) and its mutants Δ*recA* (lanes 1), Δ*recA*-*pafBC^CRISPRi/dCas9^* (lanes 2), and *pafBC^CRISPRi/dCas9^* (lanes 3) were determined by Western blot analysis with rabbit antibodies raised against RecA and LigA of *M. tuberculosis*, respectively. The control and treated MMC samples are marked by rectangles. For each lane, 24 μg of total proteins was loaded. M—Color Prestained Protein Standard, PageRuler Broad Range (Thermo Fisher Scientific, Waltham, MA, USA).

Furthermore, we examined whether the depletion of PafBC affects the overproduction of RecA at the protein level under treatment of tubercle bacilli with mitomycin C. RecA rabbit serum antibodies (see Section 2) and control LigA antibodies [19] were used to estimate the protein levels in the wild-type and *Mtb* mutant strains exposed or not to MMC (Figure 2). As expected, RecA was not immunodetected using α-RecA antibodies in *Mtb* Δ*recA-* and Δ*recA*-*pafBC*^CRISPRi/dCas9^ mutants. On the other hand, in the presence of MMC, RecA was overproduced to the same extent in the wild-type strain and mutant devoid of PafBC.

### 3.3. Removal of RecA Recombinase Leads to Transcriptional Repression of LexA in Response to the DNA Damage-Inducing Agent Mitomycin C

**Figure 3:**
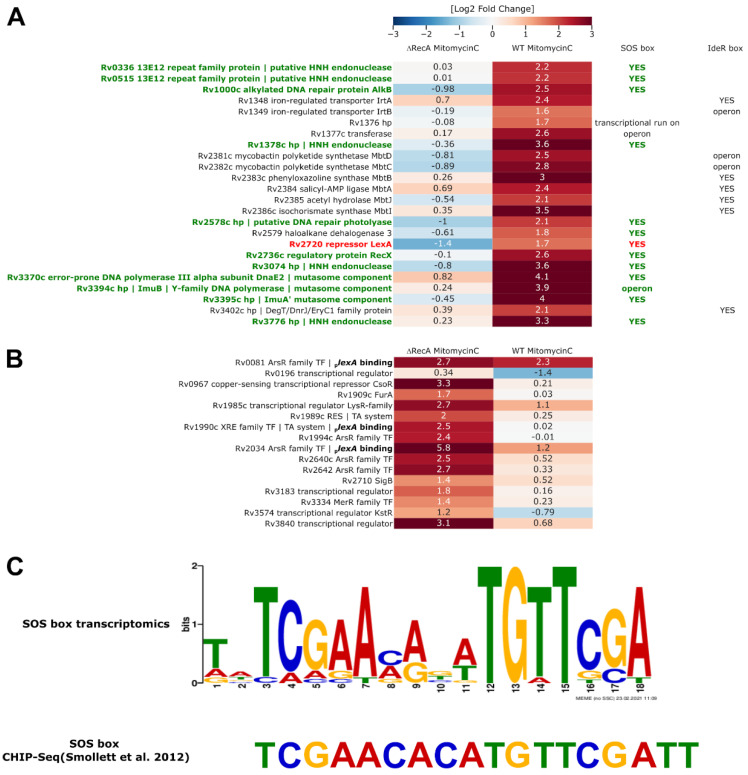
Removal of RecA leads to persistent repression of LexA- and IdeR-regulated genes, otherwise overproduced in response to DNA damage caused by mitomycin C treatment. (**A**) Heatmap of genes unresponsive to MMC treatment in the Δ*recA* strain belonging to the LexA and putative IdeR regulons and (**B**) transcriptional regulators differentially expressed in this strain relative to wild-type *H37Rv* treated with MMC. Binding to the LexA promoter (bold labels in **B**) is based on the work of Minch et al. [33] Log2-fold change values were visualized with the help of the Phyton Seaborn package and necessary dependencies. The total RNA sequencing results were obtained from three independent replicates for each strain and condition tested. Corresponding transcriptomics data were derived from the Degust automated differential expression calling platform. Genes encoding proteins relevant to DNA replication and repair are labeled in green. (**C**) MEME calculated consensus of the LexA regulatory motif—the SOS box—based on the analysis of the promoter regions of genes that changed in the Δ*recA* transcriptomics profiles.

While the RecA protein is known to play pivotal and pleiotropic functions inside bacterial cells, it is not essential for growth under stress-free conditions; moreover, mutants lacking RecA altogether can be generated in mycobacteria, including *M. tuberculosis* [15,29]. Previous studies have documented the consensus of the LexA motif in mycobacteria with ChIP-Seq methodology [30] and have revealed the transcriptomic profiles of the bacteria with the microarray approach [31,32]. In this work, we attempted to profile the whole transcriptome of the *Mtb* H_37_Rv Δ*recA* mutant by total RNA sequencing from cells cultured in the presence of a low dose of mitomycin C (5 ng/mL). Under such conditions, we found 187 genes that changed three times or more |Log_2_FC ± 1.585; *p* < 0.05|, with 65 genes being downregulated and 122 genes being overexpressed in the Δ*recA* strain compared to the wild-type strain treated with MMC (Supplementary Table S5). When confronting the results with the untreated wild-type controls, the expression of 24 genes out of 65 that were found to be downregulated under MMC treatment was at a very low level in the absence of MMC. Thus, their expression was turned on specifically in the presence of a DNA-damaging agent in a RecA-dependent fashion (Figure 3A).

Among these genes, we found some known and putative DNA damage repair factors, including the mutasome components *imuA’*, *imuB,* and *dnaE2*; alkylated DNA repair protein *alkB*; DNA repair photolyase; *recX* protein; five putative HNH endonucleases; and, importantly, the *lexA* repressor itself. The motif-based sequence analysis (MEME search) of the entire set of 187 genes that differed between the wild-type and the Δ*recA* mutant strain in response to MMC revealed the presence of the SOS box in front of 15 genes. The 16th gene that changed significantly under such conditions, namely, *rv1376*, is considered to be due to a transcriptional run-on from the expression of neighboring genes expressed on the opposite DNA strand [31]. The promoter regions predicted by MEME contained a total of 15 SOS boxes, including 3 (out of 4 known) boxes clustered in the promoter region of the LexA gene. Importantly, all remaining genes that were repressed in the Δ*recA* strain and were not regulated via SOS-box-mediated repression belonged to the IdeR regulon (Figure 3A) [34,35]. The SOS box consensus found in our study strongly agrees with previously published evidence (Figure 3C). In the set of genes that were overexpressed in the Δ*recA* mutant strain in response to mitomycin C, we found nine transposases and a resolvase. In addition to LexA, we identified 11 transcription factors whose expression differed between the wild-type and Δ*recA* strains treated with MMC (Figure 3B). Transcriptional repressors of the ArsR family were particularly overrepresented, with four ArsR paralogs strongly overexpressed in the Δ*recA* strain treated with MMC. It is important to note that the DNA-binding domains of the ArsR transcription repressors are very closely related to that of the LexA regulator and that the recently published consensus of the ArsR DNA-binding motif for *E. coli* [36] differs very little from the LexA DNA binding consensus in mycobacteria (Supplementary Figure S3). To gain a better understanding of the transcriptional regulation of LexA expression we have looked into the transcriptional regulatory networks available in the scientific literature. Based on the CHIP-seq data [33], besides LexA, six transcription factors are reported to bind in the vicinity of the *lexA* promoter region: Rv0081, Rv1049, Rv1353c, Rv1990c, Rv2034 and Rv2324. Out of these, we found Rv0081 to be overexpressed in both the wild-type and Δ*recA* mutant strains treated with mitomycin C. However, an effector transcription factor—Rv3334, whose expression is coupled with Rv0081 [37], was only overexpressed in the Δ*recA* strain treated with the DNA-damaging agent. Although it did not pass the threshold set in our transcriptomics analysis, being upregulated 2.7 times, rather than three times more than the wild-type level, multiple elements of its regulon, including numerous transcription factors, were overexpressed in the Δ*recA* strain. Among the transcription factors known to be upregulated by the Rv3334 [37] we could find Rv1990c, Rv2034, Rv2640c, and Rv2642. Rv1990c and Rv2034 were also predicted to bind to the *lexA* promoter region based on the previously mentioned ChIP-Seq data [36]. When we have looked at the ChIP-Seq coordinates, Rv0081 and Rv2034, both belonging to the ArsR family, likely bound to the very same site in the close proximity of the annotated SOS box. Thus, even in the absence of the LexA repressor, the overexpressed ArsR regulators could potentially occlude some of the SOS boxes on the DNA and prevent the expression of LexA-regulated genes and/or LexA, acting as feedback loops.

Although a large number of genes related to DNA repair remained repressed in the Δ*recA* strain treated with mitomycin C, we could find a few instances where DNA repair genes were overexpressed. Among the overexpressed genes, we found *ssb*, putative *recB*-like exonuclease *rv2119,* and putative DNA damage-inducible helicase *rv2024c* (Supplementary Table S5).

### 3.4. Whole-Cell Proteomics Profiling Confirms the Downregulation of the LexA Repressor in Cultures of Both Wild-Type and ΔRecA Mutant Strains Caused by RecA-Dependent Coproteolysis and Transcriptional Repression, Respectively

**Figure 4:**
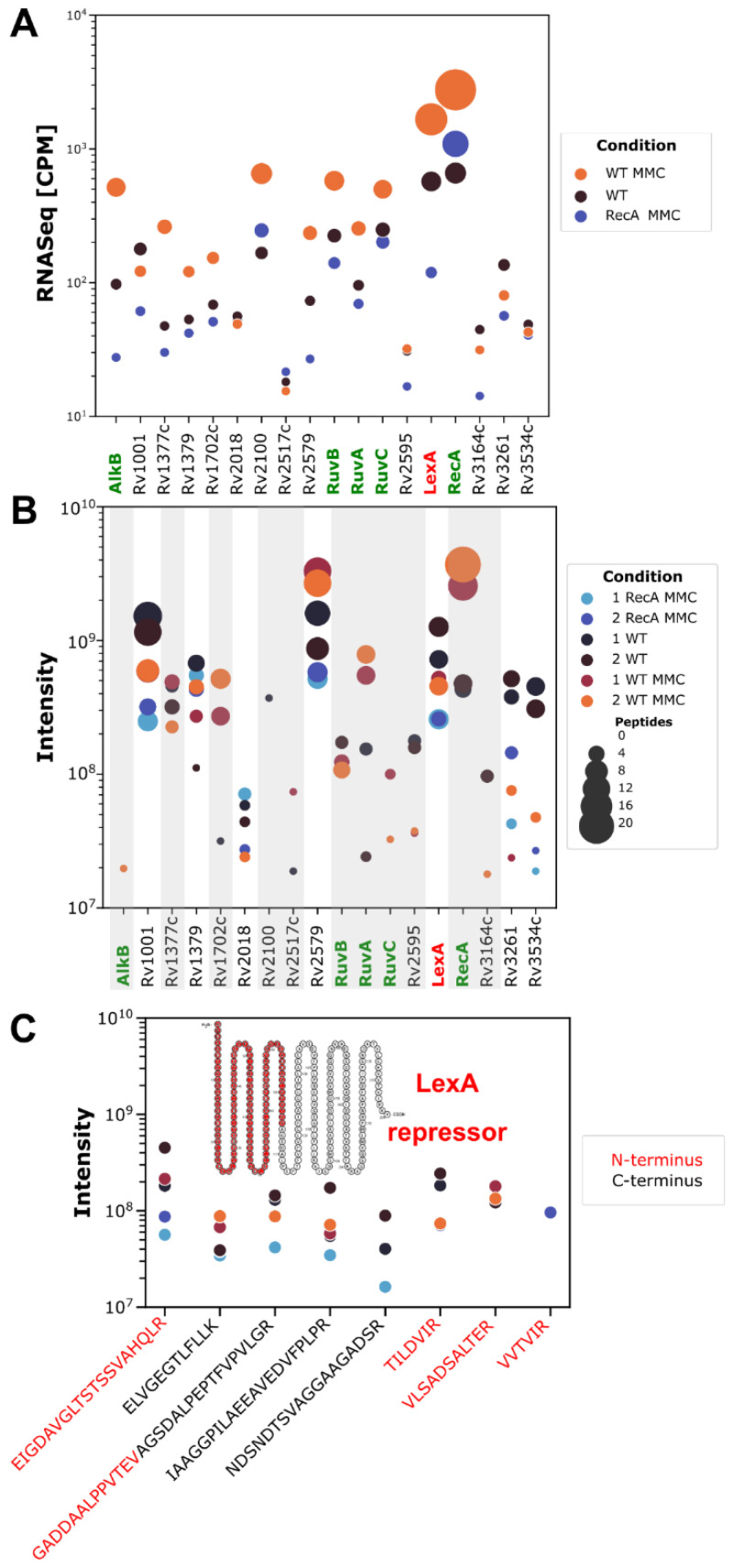
Proteomics profiles of *Mycobacterium tuberculosis* strains cultured in the presence of mitomycin C confirm the downregulation of proteins whose expression is regulated via the SOS box in the Δ*recA* strain, including LexA itself. Total RNA-Seq mappable reads were converted to CPM and plotted on the logarithmic scale (**A**). MaxQuant-derived protein intensities (**B**) for proteins belonging to the putative LexA regulon are plotted for comparison of their levels from untreated and MMC-treated wild-type and ΔrecA strains, respectively. Data were obtained from high-performance mass spectrometry analysis of whole-cell protein lysates analyzed on a Q exactive mass spectrometer (Thermo Fisher Scientific) from two biological replicates for each tested strain and condition. Gray background was applied on the protein intensity plot, if a given protein was not detected in the RecA mutant strain, to pinpoint the most significant changes. RecA—Δ*recA* strain, WT—wild-type *M. tuberculosis*, MMC—mitomycin C. (**C**) Intensities of individual peptides of the LexA regulator are presented with respect to their localization in the DNA-binding portion (red labels) or peptidase portion (black labels) of the transcriptional repressor.

We sought to confirm whether the LexA transcriptional downregulation seen in the Δ*recA* mutant strain was also noticeable at the protein level. We isolated the total protein extracts from relevant mycobacterial strains using buffers enriched in urea and SDS to ensure maximal protein liberation in the resulting lysates. We then submitted such preparations to high-performance mass spectrometry analysis to obtain whole-cell proteomics profiles. While the obtained proteomics spectra allowed for identification of a very high number of proteins, resulting in over 2000 identifications in each sample (more than half of the entire proteome), the elements of the SOS regulon were not highly represented. Out of 38 putative SOS-box-regulated genes (combined information from individual genes based on Smollett et al. [30] and Davis et al. [38] and their predicted operons), 18 proteins were abundant enough to be detected by mass spectrometry, with putative AlkB (Rv1000c) and RuvC (Rv2594c) only detectable following mitomycin C treatment (Figure 4B).

Protein intensity analysis performed on spectrometric data with MaxQuant software revealed that the RecA mutant was indeed devoid of RecA protein. The wild-type as well as the ΔrecA strain overproduced metallothionein Rv0185A to a very high level, proving that the metal scavenging is important for DNA damage response. This was the most relevant change on the proteomic level for both strains treated with mitomycin C (Supplementary Table S6). The absence of key Holliday junction resolvase components, namely, RuvA, RuvB and RuvC, was noticeable in the Δ*recA* strain. These proteins were otherwise overproduced in the wild-type upon treatment with mitomycin C based on transcriptional profiling as well as mass spectrometry (Figure 4A,B), and all were regulated by an SOS box within their respective promoters. In contrast, two other DNA repair proteins were overproduced in the absence of RecA: UvrA and an essential protein SSB (Supplementary Table S6). The expression of these two proteins is not dependent on the presence of a functional RecA protein in *M. tuberculosis*.

In agreement with our transcriptional profiling data, mycobacteria devoid of RecA and exposed to mitomycin C produced less LexA protein, compared to the wild-type strain also treated with the compound (Figure 4A,B).

Since the LexA protein should undergo RecA-induced autoproteolysis [39], individual peptides belonging to the *N*-terminus, *C*-terminus and cleavage regions were estimated and their corresponding levels were found to be similar for wild-type and Δ*recA* strains treated with MMC. On average, they were lowest in the Δ*recA* strain (Figure 4C). Under such conditions, the LexA regulon should be (at least partially) derepressed along with depletion of the repressor, which is similar to the results of RecA-dependent proteolysis of LexA. In contrast, the genes under regulation of SOS boxes remained transcriptionally silent, which could result from binding of the ArsR repressors to the SOS boxes in the absence of LexA or other phenomena. The drop in the LexA level observed in our analysis was about five to ten times less than the wild-type, which could be insufficient to trigger SOS response. Overall, our findings may be indicative of a previously overlooked complexity of the SOS box-dependent transcriptional regulation of DNA damage repair-related genes.

### 3.5. PafBC-Dependent Regulation of Gene Expression in Response to Mitomycin C Is Conserved among Saprophytic and Pathogenic Mycobacteria

The recently identified PafBC transcriptional regulator adds an additional level of complexity to the landscape of the bacterial response to DNA-damaging agents [13,14]. Based on recent work on *M. smegmatis*, RecA/LexA and PafBC are now considered the two main factors regulating the response of mycobacteria to the genotoxic activity of mitomycin C. We noticed that the removal of RecA alone caused a much more pronounced response to DNA damage, with transcripts for 346 genes accumulating to significant levels upon mitomycin C treatment compared to the untreated wild-type *Mtb* H_37_Rv strain. The expression of 147 of these genes was downregulated in the Δ*recA PafBC^CRISPRi/dCas9^* strain, and the initial MEME analysis revealed the presence of a putative PafBC-binding motif in the promoter regions of 38 of these genes. PafBC is considered a positive regulator of gene expression, and consistent with that hypothesis, PafBC-driven overexpression was apparent in response to MMC. The motif was often found to span the −35 and −10 promoter sequences but was occasionally present just downstream of the transcription start site, judging by the RNA sequencing traces. The PafBC DNA motif seems slightly more degenerated in *M. tuberculosis* than in *M. smegmatis*, thus allowing substitutions of TGTCGG-10xN-TA-3xN-T to TGTCAG-10xN-TA-3xN-T or TGTCAC-10xN-TA-3xN-T (Supplementary Figure S4). Genome-wide FIMO analysis of promoter regions with these three variations of the PafBC-binding motifs revealed the presence of a large number of genes putatively regulated by PafBC (Supplementary Table S7). Supported by the RNA sequencing results, the presence of 75 putative motifs in gene promoter regions was associated with PafBC-driven overexpression of 126 genes in response to mitomycin C (Supplementary Table S7).

**Figure 5:**
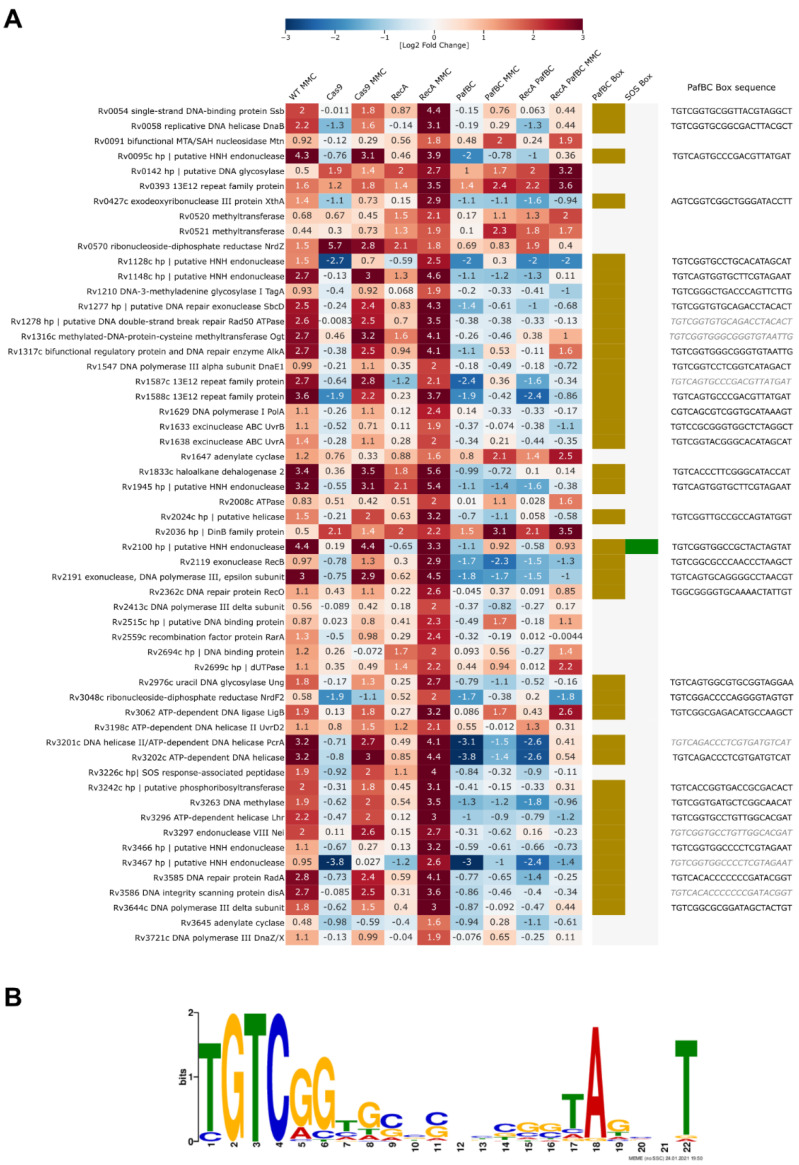
PafBC transcription factor drives the expression of the majority of genes responsive to mitomycin C treatment in *Mycobacterium tuberculosis*. (**A**) Heatmap summarizing changes seen in transcriptomics profiles of genes upregulated during MMC treatment of Δ*recA* relative to untreated wild-type *H37Rv*. Only transcripts encoding proteins relevant to DNA replication and repair are shown. The presence of a putative PafBC regulatory motif is indicated, and the corresponding sequence is provided for each PafBC-dependent gene. Sequences for genes coregulated within the same operons are grayed out. Corresponding transcriptomics data were derived from the Degust automated differential expression calling platform. All sequencing results were obtained from three independent biological replicates. (**B**) *M. tuberculosis* PafBC DNA-binding motif consensus is provided based on the MEME analysis of the promoter sequences of genes encoding DNA repair factors listed in the (**A**) panel of the figure.

PafBC-driven overexpression was clearly enhanced in the Δ*recA* strain treated with MMC; hence, this strain served best for the RNA-Seq-based prediction of the PafBC regulon. Fifty-six overexpressed genes were related to DNA replication and repair in this strain, and 38 of these had PafBC motifs present within their promoter regions (Figure 5A) with the abovementioned consensus (Figure 5B).

### 3.6. Transcriptional Profiling and Proteomics Analysis Revealed Few DNA Damage Repair Genes That Were Overexpressed in Response to DNA Damage When Both the PafBC and RecA/LexA Regulatory Networks Were Tuned Down

Transcriptomics profiles revealed that the gene encoding the error-prone DNA polymerase *dnaE2* was still significantly upregulated in the Δ*recA-pafBC^CRISPRi/dCas9^* strain, although clear RecA dependency was noted in the single Δ*recA* mutant. *DnaB* helicase, on the other hand, also passed the 1.583 threshold and was significantly overexpressed in the double mutant but partially dependent on PafBC regulation. The operon encoding AlkA and Ogt DNA repair factors was also upregulated specifically in response to MMC treatment in all tested strains, including the Δ*recA-pafBC^CRISPRi/dCas9^* strain. Interestingly, the overexpression of all the above-listed genes was associated with the presence of a putative upstream SOS box. This could have resulted from the downregulation of LexA to the level where it no longer represses the DNA repair genes. Although this explanation seemed plausible, it was not observed for all SOS-box-regulated genes, with the example being the *imuA’-imuB* operon (Supplementary Figure S5). Additionally, the level of expression of the *alkB* gene remained at the wild-type level and was responsive to induction by MMC.

Proteomics profiling of Δ*recA* and *pafBC^CRISPRi/dCas9^* mutant strains revealed that a relatively large fraction of proteins changed in response to MMC in *pafBC^CRISPRi/dCas9^*. Interestingly, the putative AlkB protein was only detectable in the lysates of the double mutant treated with MMC. Similar to the single Δ*recA* strain, the Holliday junction resolvase RuvC protein disappeared from the mass spectra of the *pafBC^CRISPRi/dCas9^* strain. The Holliday junction resolvase RuvX was overproduced in all strains following MMC treatment (Supplementary Table S6). Surprisingly, the expression of its gene was practically unchanged in the corresponding transcriptomic profiles; therefore, the regulation of its expression may be at the posttranscriptional level. Many of the downregulated proteins belonged to the DosR regulon, thus confirming the RNA-Seq results, where similar observations were apparent. It is worth noting that the experiments were performed independently, not from the same cultures, but grown separately, and the same findings were observed in two independent omics platforms. This result is indicative of true DosR downregulation in response to MMC treatment or possibly DNA damage in general.

## 4. Discussion

The growing incidence of drug- and multidrug-resistant tuberculosis indicates that tuberculosis may become an incurable disease once again. Discovery of novel antituberculosis drugs and studies related to the mechanisms of acquiring drug resistance are recognized as priorities in tuberculosis research. Collective scientific evidence suggests that mycobacterial drug resistance and mutagenesis are controlled by the central DNA recombinase RecA [11]. RecA recombinase plays pleiotropic roles in DNA recombination and repair. This ubiquitous protein, which is found in all cellular organisms, is primarily involved in homologous recombination. It is the key enzyme mediating DNA strand exchange reactions during recombination. In the presence of the single-stranded fragment of the DNA (ssDNA), e.g., following DNA damage, it coats the ssDNA and forms filaments that later engage in mediating base pairing with the homologous double-stranded DNA duplex [40]. The protein possesses ATP hydrolase activity; however, this activity is believed to help with RecA filament progression and RecA recycling rather than contributing to the DNA strand exchange reaction itself [41]. The deletion of RecA causes severe sensitization of the cell to DNA damage, which is mainly triggered by so-called “reckless DNA degradation,” following DNA damage, via extensive DNA resection driven by RecBCD and similar exonuclease complexes [42]. In contrast, under normal circumstances, RecBC or RecBCD protein complexes are regarded as loading factors for RecA protein, and they load this protein preferentially onto the 3′-ended DNA strand of resected DNA. RecA competes for ssDNA binding but also cooperates with other ssDNA-binding proteins, such as SSB. While SSB is initially required to melt any secondary structures that may be present in the single-stranded DNA fragment, it is later easily displaced by the growing RecA filament in preparation of DNA strand exchange [43]. We observed a significant overexpression of SSB in the Δ*recA* mutant strain regulated by PafBC, which could increase the stability or lifespan of single-stranded DNA in the absence of RecA. PafBC also upregulated the AdnAB repair complex (Supplementary Figure S3B), which is the major processive helicase mediating RecA-dependent homologous recombination in mycobacteria [9], and the sole RecO gene from another RecA-dependent system, RecFOR. However, Gupta and coworkers showed that mycobacterial RecO can be involved in RecA-dependent HR as well as in RecA-independent single strand annealing [44]. In contrast, the RecBCD complex involved in single strand annealing was not induced during MMC treatment, and its expression nearly reached significant depletion in the Δ*recA* strain treated with MMC. Similarly, the expression of the NHEJ proteins Ku and LigD was downregulated in response to the MMC treatment of mycobacterial cells. No putative regulatory elements were found in the promoters of the RecBCD and LigD and Ku operons (Supplementary Table S5).

In addition to its central role in the mediation of DNA strand exchange during homologous recombination, the RecA protein is also a regulatory protein and acts as a coprotease for proteins undergoing autoproteolysis. Phosphorylation of serine 207 has recently been linked to the regulatory activities of RecA [11]. In the same study, mycobacterial protein coprotease activity but not ATP hydrolysis or DNA strand exchange activities was shown to be inhibited by cardiolipin from the inner cell membrane. The report suggests that the RecA recombination factor associates with the cell membrane after DNA damage has been repaired. Another recent study noted that RecA pupylation plays a critical role in the clearance of RecA as well as some other DNA damage repair factors via the mycobacterial proteasome [14].

**Figure 6:**
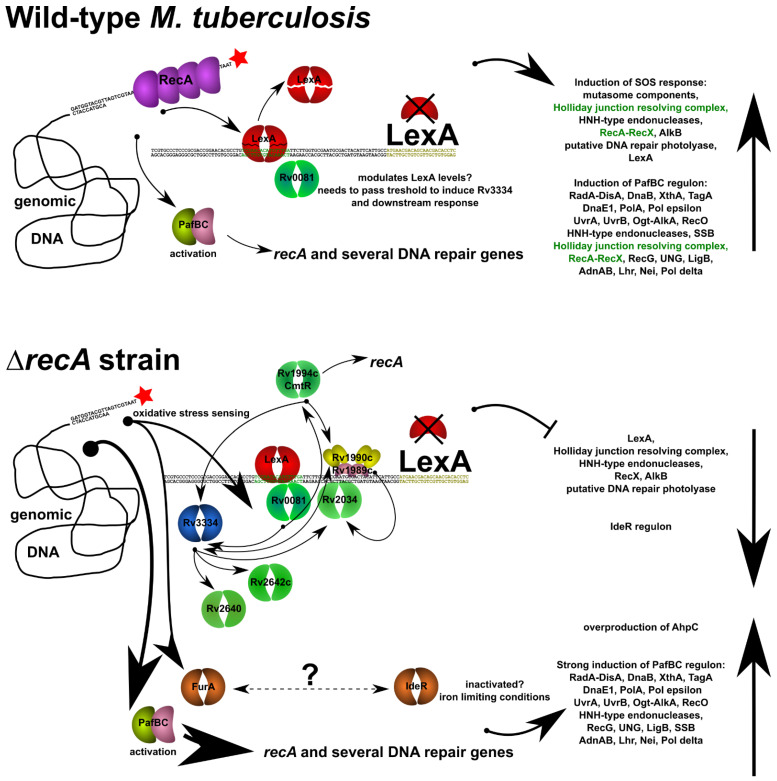
A proposed model for the regulation of SOS response and associated DNA damage responses in *M. tuberculosis.* Regulatory network influencing the expression levels of LexA is likely triggered by the Rv0081 transcriptional regulator involved in stress sensing. Removal of RecA might cause additional stress to the cell, which leads to more pronounced overexpression of Rv0081. Upon passing a certain threshold, Rv0081 induces expression of an effector regulator Rv3334 [37]. In turn, Rv3334 promotes the expression of a large number of transcription factors modulating the downstream response. Rv0081 as well as four downstream regulators: Rv2640, Rv2642c, Rv2034, and Rv1994c belong to the ArsR family of transcriptional regulators, suggesting there is a possible link between this group of transcription factors and DNA damage response. Removal of RecA increases the efficiency of PafBC activation, which causes more pronounced overexpression of numerous DNA repair genes in the absence of RecA. Iron sensing transcription factors are also likely to contribute to the response in the Δ*recA* background, since overexpression of *furA* correlates with strong overexpression of *ahpC* in this strain. On the other hand, the IdeR regulon remains unresponsive to DNA damage in this strain, unlike in the wild-type treated with mitomycin C.

RecA along with the LexA transcriptional repressor are key factors involved in the bacterial SOS response, which is a general, inducible transcriptional response to DNA damage. UV irradiation, genotoxic agents and some classes of antimicrobial drugs are known to cause the induction of the SOS system. The SOS response is a result of RecA activation, which induces cleavage of the LexA transcriptional repressor occluding SOS boxes, or DNA motifs located within the promoters of genes relevant to DNA repair, because of its coprotease activity. Initial experiments based on microarray RNA profiling revealed 16 functional LexA-binding sites in the *M. tuberculosis* genome [31]. Subsequent ChIP-Seq analysis confirmed the binding of RecA to all 16 boxes and added more binding sites for a total of 25 functional SOS boxes on the bacterial chromosome [30]. The genotoxic agent mitomycin C is commonly used as a specific and well-characterized inducer of SOS responses in a variety of bacteria, including mycobacteria. Upon depletion of RecA and PafBC transcriptional regulators, we started observing the expression of some DNA repair proteins from SOS-box-regulated promoters (previously annotated SOS box or putative, which were discovered by us via genome-wide FIMO analysis) (Supplementary Figure S5). This phenomenon was observed in the case of the error-prone DNA polymerase *dnaE2,* an operon encoding alkylated DNA repair factors *alkA-ogt* and the replicative DNA helicase *dnaB*. This finding could be a result of the transcriptional silencing of LexA expression, which was deepest in the Δ*recA pafBC^CRISPRi/dCas9^* strain and could lead to derepression of some of the LexA-controlled promoters, for which the protein has weaker affinity. On the other hand, we observed a broad response of various transcription factors, particularly from the ArsR family of transcriptional repressors. It has been recently shown that *E. coli* ArsR [35] is able to bind the DNA motif with a consensus closely resembling the mycobacterial SOS box (Supplementary Figure S3). We speculate that the repression of LexA transcription upon the removal of RecA and even stronger repression in the double mutant strain was driven by ArsR-mediated repression, and this observation correlates very well with the increasing accumulation of ArsR factors in the investigated strains. A transcription trace analysis indicated that although the PafBC motif is present in the vicinity of LexA orf, it is not involved in the regulation of its expression. First, the direction of the PafBC box is opposite to that of the LexA gene, and we have seen that the directionality of the PafBC motif is essential to its functionality. Second, the expression of the LexA gene seems to be regulated via the sole SOS box (Supplementary Figure S6), which is consistent with the literature [45]. We confirmed in vivo that the other three SOS boxes and the PafBC-binding motif were involved in the regulation of the expression of the operons encoding the Rv2019c, Rv2018c and Rv2017c proteins and did not influence the expression level of the LexA transcript. In addition to ArsR family regulator overexpression, we observed that removal of RecA affected the IdeR regulon equally strongly as it did the LexA regulon. IdeR is an important factor regulating the oxidative stress response in actinomycetes, and its removal was previously shown to sensitize bacteria to toxic H_2_O_2_ damage [46]. It is required for iron homeostasis and considered an essential indispensable virulence factor of *M. tuberculosis* [47]. FurA, another iron sensing response regulator was also overexpressed in the Δ*recA* mutant. The overexpression of *furA* was associated with strong overexpression of the alkylhydroperoxide reductase AhpC, important for oxidative damage defense. The *ahpC* gene was previously shown to be regulated by FurA in *M. smegmatis* [48], which alike *M. tuberculosis* lacks a functional ortholog of OxyR. The transcription factor overexpression studies [49] along with comprehensive ChIP-Seq analysis [36] and database searching (http://networks.systemsbiology.net/mtb/) allowed us to propose a model of the regulation of SOS response in pathogenic mycobacteria (Figure 6).

Together, we were able to carefully excerpt protein members involved in the response of the deadly *M. tuberculosis* to the model genotoxic agent mitomycin C. Using total RNA sequencing coupled with bioinformatics analyses, we were able to clearly correlate the presence of regulatory elements that drive the RecA-dependent and RecA-independent responses to DNA damage. The transcriptional profiles were correlated with proteomics profiles obtained from high-performance mass spectrometry. With the currently updated gene annotation, individual changes in the DNA replication and repair machinery under the tested conditions can be better understood. It is critically important to understand the regulators and effectors of the SOS response because they are considered a likely link to increased mutation rates and persister cell formation, thereby contributing to the drug resistance of *M. tuberculosis* treated with novel antituberculosis chemotherapeutics, such as moxifloxacin [50].

The analysis of evolutionary pressure showed the fundamental role of *recA* across all kingdoms of life. *M. tuberculosis recA* is highly conserved, with the lowest number of accumulated mutations per 100 bp among all 42 genes analyzed to date within the virtual database [27,51]. The SOS repressor LexA is more restricted in the phylogenetic tree and was detected only in bacteria and episodically in species of Viridiplantae and Opisthokonta. It remains to be established whether eukaryotic proteins similar to LexA are true homologs or whether they arose due to convergent evolution with no functional or structural preferences [52]. In turn, representative proteins of the PafBC system are more scarcely spread across the tree of life and show less overall similarity within the population of clinical strains of *M. tuberculosis*.

In summary, we show that *M. tuberculosis* response to mitomycin C is carried out by multiple factors, controlled by a complex interplay between LexA, PafBC and other regulators, which all contribute to the overall ability of the pathogen to adequately respond to DNA damage.

## Data Availability

The data presented in this study are available online (see supplementary files) or upon request from the corresponding author.

## Supplementary Materials

The following are available online at https://www.mdpi.com/article/10.3390/cells10051168/s1: Figure S1. Co-occurrence of DNA repair genes, Figure S2. Alignment of mycobacterial RecA, PafB and PafC proteins, Figure S3. Comparison between the MEME-derived DNA binding consensus of the *M. tuberculosis* LexA and the DNA binding consensus reported for *E. coli*’s ArsR regulator (A). Clustal Omega alignment of *E. coli* and *M. tuberculosis* ArsR orthologues (B), Figure S4. Zoom in at the genomic location of putative PafBC DNA binding motifs within the promoter regions of chosen DNA repair genes, Figure S5. Transcriptional traces of DNA repair genes overproduced in response to MMC treatment in the ΔrecA-pafBC^CRISPRi/dCas9^ strain, associated with the presence of putative SOS-box-like motifs (A,B) and an unresponsive control (C), Figure S6. Transcriptional traces of the LexA region indicate that PafBC is not involved in the regulation of lexA expression but the rv2719c operon instead in agreement with its directionality, Table S1. Bacterial strains, Table S2. Plasmids and primers, Table S3. Variability and dN/ds. of DNA repair genes *M. tuberculosis*, Table S4. SNP variation of DNA repair genes of *M. tuberculosis*, Table S5. Genes differentially expressed between H37Rv and ΔrecA strains cultured in the presence of 5 ng/mL mitomycin C, Table S6. MaxQuant proteomics quantification for whole cell lysates obtained from mycobacterial strains cultured in the presence of 5 ng/mL mitomycin C, Table S7. Genes differentially expressed between ΔrecA and ΔrecA, pafBC^CRISPRi/dCas9^ strains cultured in the presence of 5 ng/mL mitomycin C with FIMO predicted PafBC regulatory motif within their promoter regions.
