# Supplementary material for: Dissecting the RecA-(In)dependent Response to Mitomycin C in Mycobacterium tuberculosis Using Transcriptional Profiling and Proteomics Analyses
Source: Cells. 2021 May 11;10(5):1168. doi: 10.3390/cells10051168 (PMC8151990; doi:10.3390/cells10051168)
Supplement: Supplementary file 1 [file cells-10-01168-s001.zip › Supplementary files Figures-Tables.pdf]

Dissecting the RecA-(In)dependent Response to Mitomycin C in *Mycobacterium tuberculosis* Using Transcriptional Profiling and Proteomic Analyses

Anna Brzostek, Przemysław Płociński, Aneta Ciszewska, Filip Gąsior, Alina Minias, Jakub Pawełczyk, Bożena Dziadek, Dominik Strapagiel and Jarosław Dziadek

Supplementary data:

Figure S1. Co-occurrence of DNA repair genes.

Figure S2. Zoom in at the genomic location of putative PafBC DNA binding motifs within the promoter regions of chosen DNA repair genes. Transcription traces from the RNA Seq experiments are shown above the relevant DNA sequences of the promoter regions of *ruvC* (A), *uvrD* (B) and *radA* (C) genes.

Figure S3. Comparison between the MEME-derived DNA binding consensus of the *M. tuberculosis* LexA and the DNA binding consensus reported for *E. coli*'s ArsR regulator (A) [35]. Clustal Omega alignment of *E.coli* and *M. tuberculosis* ArsR orthologues (B).

Figure S4. Transcriptional traces of DNA repair genes overproduced in response to MMC treatment in the Δ*recA-pafBC*$^{CRISPRi/dCas9}$ strain, associated with the presence of putative SOS-box-like motifs (A,B) and an unresponsive control (C).

Figure S5. Transcriptional traces of the LexA region indicate that PafBC is not involved in the regulation of *lexA* expression but the *rv2719c* operon instead in agreement with its directionality. The expression of LexA is repressed in the absence of RecA coprotease activity in mycobacterial cell.

Table S1. Bacterial strains.

Table S2. Plasmids and primers.

Table S3. Variability and dN/ds. of DNA repair genes *M. tuberculosis*.

Table S4. SNP variation of DNA repair genes of *M. tuberculosis* – see Excel file.

Table S5. Genes differentially expressed between H37Rv and Δ*recA* strains cultured in the presence of 5 ng/ml mitomycin C– see Excel file.

Table S6. MaxQuant proteomics quantification for whole cell lysates obtained from mycobacterial strains cultured in the presence of 5ng/ml mitomycin C– see Excel file.

Table S7. Genes differentially expressed between Δ*recA* and Δ*recA*, *pafBC*$^{CRISPRi/dCas9}$ strains cultured in the presence of 5 ng/ml mitomycin C with FIMO predicted PafBC regulatory motif within their promoter regions– see Excel file.

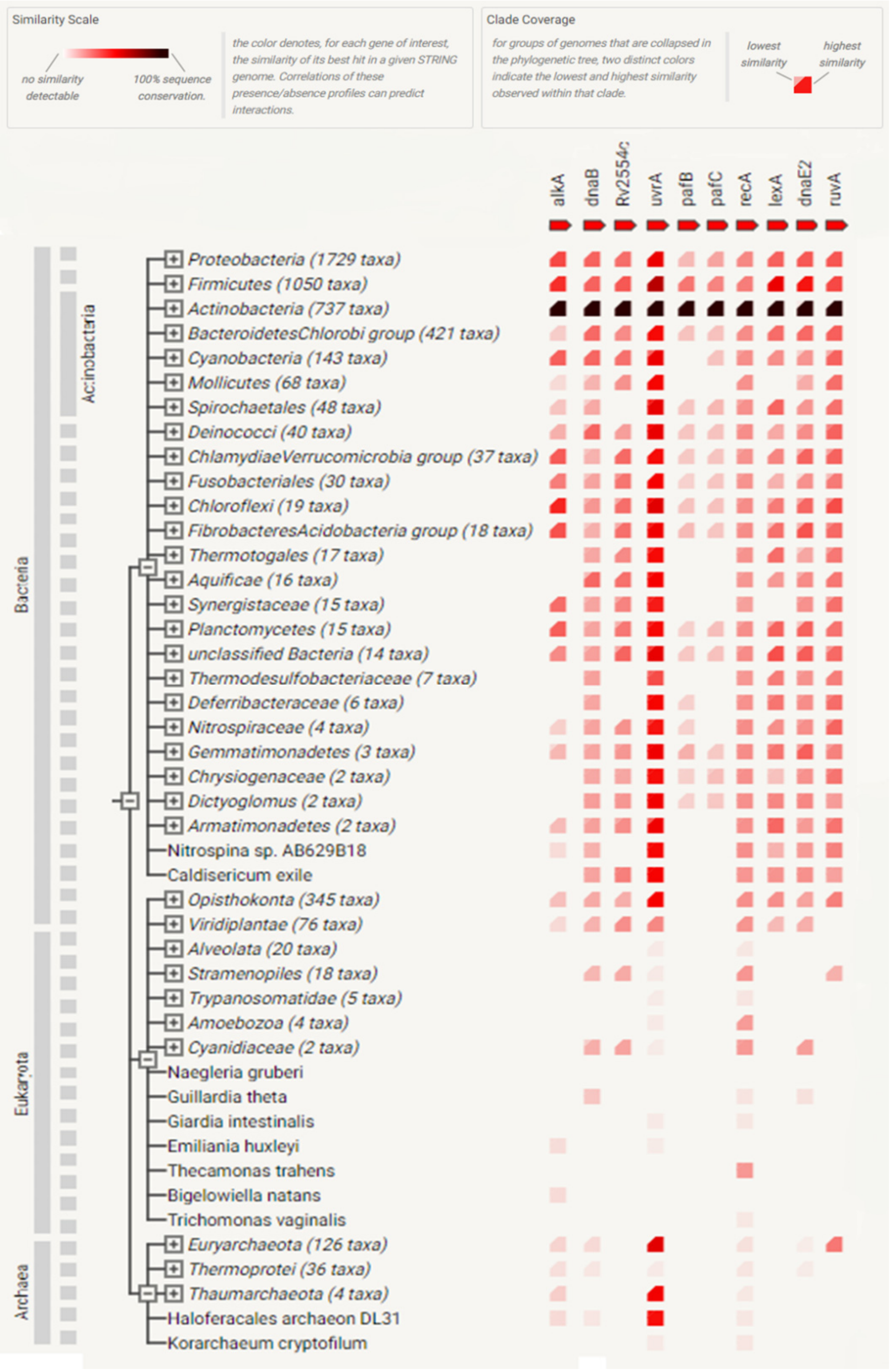

Figure S1. Co-occurrence of DNA repair genes. We performed virtual detection of gene homologs across phylogenetic tree of life. We used co-occurrence gene detection of STRING Protein software to identify homologs of *M. tuberculosis* genes.

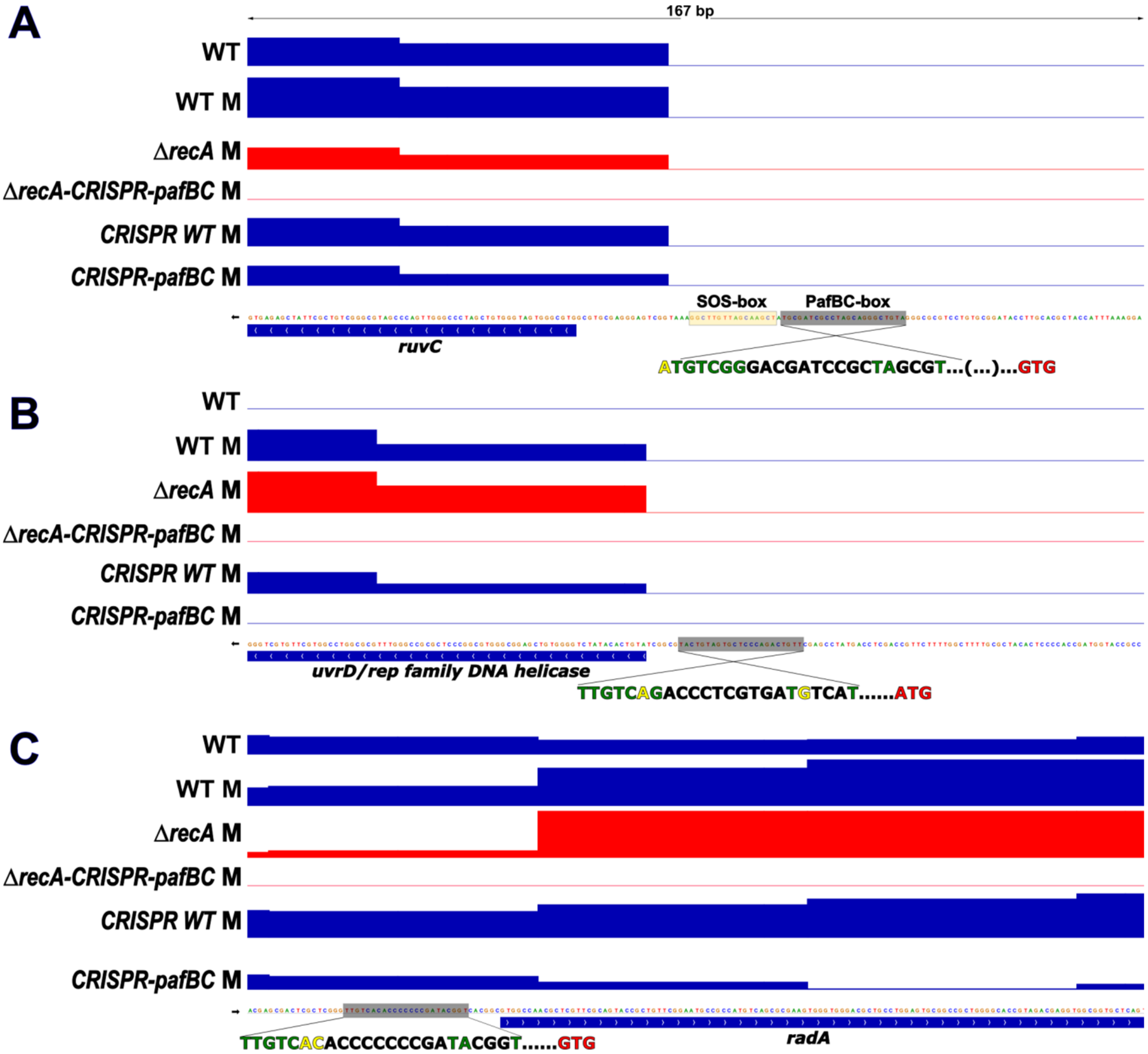

Figure S2. Zoom in at the genomic location of PafBC DNA binding motifs within the promoter regions of chosen DNA repair genes. Transcription traces from the RNA Seq experiments are shown above the relevant DNA sequences of the promoter regions of *ruvC* (A), *adnA/uvrD* (B) and *radA* (C) genes.

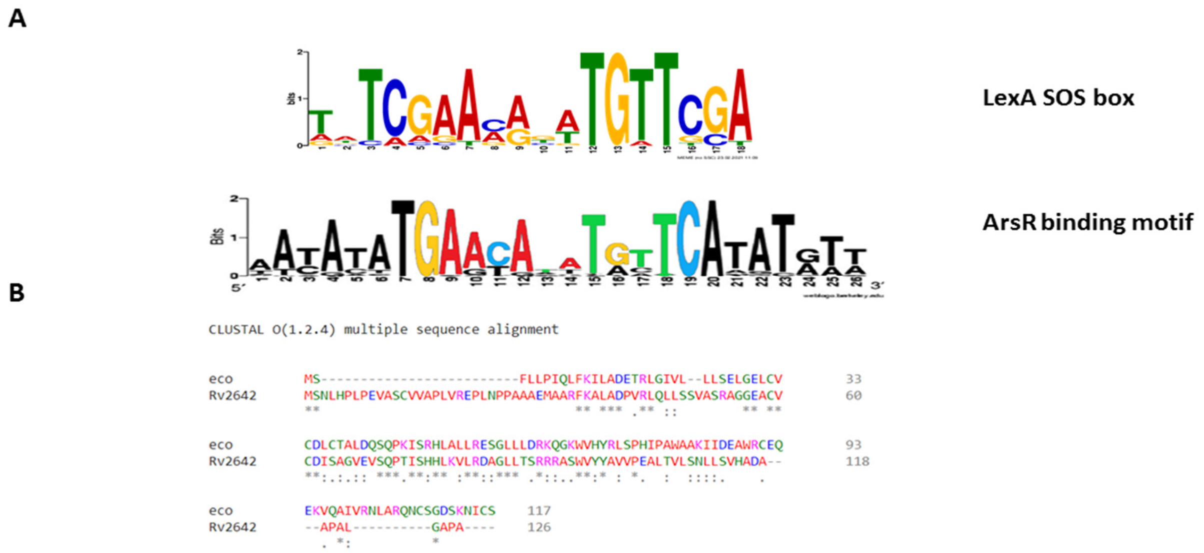

Figure S3. Comparison between the MEME-derived DNA binding consensus of the *M. tuberculosis* LexA and the DNA binding consensus reported for *E. coli*'s ArsR regulator (A) [35]. Clustal Omega alignment of *E. coli* and *M. tuberculosis* ArsR orthologues (B).

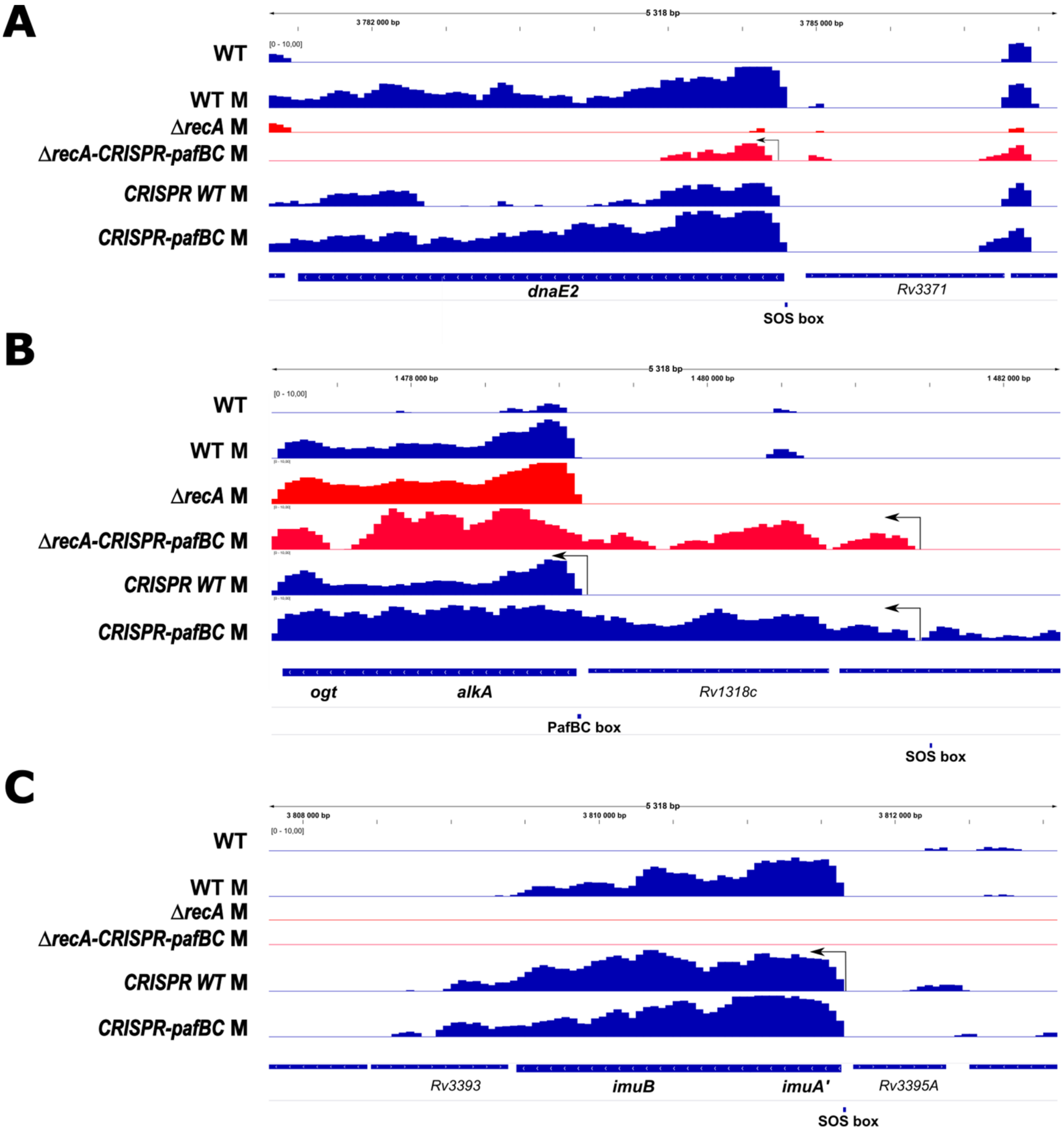

Figure S4. Transcriptional traces of DNA repair genes overproduced in response to MMC treatment in the Δ*recA*-*pafBC*$^{CRISPRi/dCas9}$ strain, associated with the presence of putative SOS-box motifs (A,B) and an unresponsive control (C).

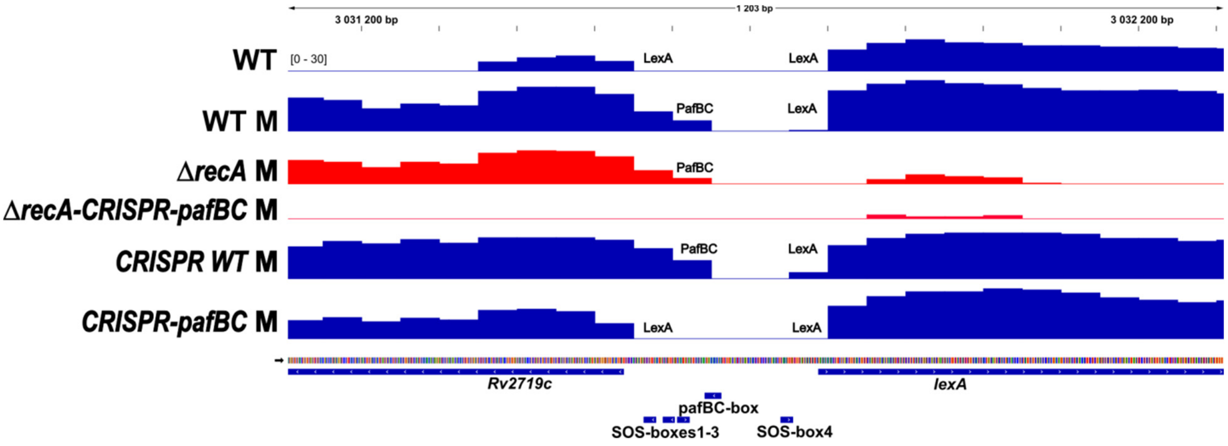

Figure S5. Transcriptional traces of the LexA region indicate that PafBC is not involved in the regulation of *lexA* expression but the *rv2719c* operon instead in agreement with its directionality. The expression of LexA is repressed in the absence of RecA coprotease activity in mycobacterial cell.

Table S1. Bacterial strains.

**Name/Genotype**	**STRAINS**	**Reference**
Top10F′	*Escherichia coli* strain	Invitrogen
Mtb H$_{37}$Rv	*M. tuberculosis* - wild type	Laboratory stock
⊗*recA*	*M. tuberculosis* defective in the synthesis of RecA	Brzostek et al., 2014
Mtb CRISPR-Cas9	*M. tuberculosis* carrying integrative pLJR965	This study
Mtb CRISPR-Cas9-PafBC	*M. tuberculosis* with inducible depletion of PafBC	This study
Mtb ⊗*recA*- CRISPR-Cas9-PafBC	*M. tuberculosis* with inducible depletion of PafBC and defective in the synthesis of RecA	This study

Table S2. Plasmids and primers.

**Plasmids Used for This Study**	**Vector type**	**Source**
pJET 1.2/blunt	Blunt cloning vector, Amp^R	Thermo Scientific
pHis Parallel	Expression vector, Ap^R	Sheffield et al.,1998
pJLR965	CRISPR-Cas9 cloning vector Km^R	Rock JM et al., 2017
**Primers Used in This Study**		
Name of oligo	Sequence (5′>3′)	Application
RvrecA-BglII-F	cagatctgATGACGCAGACCCCCGATCG	*recA* for pHis Parallel
RvrecA –EcoRI-R	cgaattctcaGAAGTCGACGGGGGCGGG	
Crispr/Cas9 -F	GATCGAGATGGCCCGCGAGA	CRISPR Cas9 recombinant strains verification
Crispr/Cas9-R	CACGGCGTGGTGGTGGTAGGT	
pafBC-sgRNA-S	GGGAACCTGCGCTCCGATCCGGGACAG	CRISPR -Cas9 PafBC recombinant strain
pafBC-sgRNA-R	AAACCTGTCCCGGATCGGAGCGCAGGT	

Table S3. Variability and dN/ds. of DNA repair genes *M. tuberculosis*.

		Essentiality In Vitro (deJesus)	Essentiality Infection	dN/ds.	Total Number of Sites	No. of Polymorphic Sites	No. of Polymorphic Sites per 100 bp	Number of Unique Sequences
alkA	Rv1317c	non-essential	non-essential	0.925940383	1491	51	3.420523139	51
dnaB	Rv0058	essential		1.026848531	2625	91	3.466666667	92
dnaE2	Rv3370c	non-essential	non-essential	0.752216517	3240	98	3.024691358	95
lexA	Rv2720	growth-defect	non-essential	0.69368535	711	29	4.078762307	30
pafB	Rv2096c	non-essential	non-essential	0.749597961	999	26	2.602602603	27
pafC	Rv2095c	non-essential	non-essential	1.19693565	951	35	3.680336488	36
recA	Rv2737c	non-essential (growth defect)	non-essential	0.862395437	2374	42	1.769165965	44
ruvA	Rv2593c	growth-defect	non-essential	0.320652067	591	19	3.214890017	19
ruvX	Rv2554c	essential		1.745289611	513	12	2.339181287	13
uvrA	Rv1638	non-essential	non-essential	0.401143688	2919	77	2.637889688	71
